# The Anti=Vivisection Bill in Congress

**Published:** 1898-12

**Authors:** D. S. Lamb

**Affiliations:** Secretary; Washington, D. C.


### Correspondence.

### The Anti-Vivisection Bill in Congress.

WASHINGTON, D. C., November 18, 1898.

*Editor Texas Medical Journal:*

At a recent meeting of the Joint Committee of Scientific Societies of this city, on Vivisection, the following resolution was adopted:

*Resolved,* That the Secretary be authorized to call the attention of the prominent medical and scientific journals of the country to the importance of the meeting of the American Humane Society to be held in this city in December, proximo, and to request that editorial notice be taken of the danger that the influence likely to be exerted at that meeting may cause the [anti-]vivisection bill now pending in the Senate to be called up and passed.

I was also directed to ask that you will advise your readers to write to their respective Senators and Representatives in regard to the matter. Yours truly,

D. S. LAMB, M. R., Secretary.

